# Impacts of Hurricanes Irma and Maria on *Aedes aegypti* Populations, Aquatic Habitats, and Mosquito Infections with Dengue, Chikungunya, and Zika Viruses in Puerto Rico

**DOI:** 10.4269/ajtmh.19-0015

**Published:** 2019-04-08

**Authors:** Roberto Barrera, Gilberto Felix, Veronica Acevedo, Manuel Amador, Damaris Rodriguez, Luis Rivera, Orlando Gonzalez, Nicole Nazario, Marianyoly Ortiz, Jorge L. Muñoz-Jordan, Stephen H. Waterman, Ryan R. Hemme

**Affiliations:** 1 Entomology and Ecology Team, Dengue Branch, Centers for Disease Control and Prevention, San Juan, Puerto Rico;; 2 Vector Control Unit of Puerto Rico, Puerto Rico Science Trust, San Juan, Puerto Rico;; 3 Molecular Diagnostic Laboratory, Dengue Branch, Centers for Disease Control and Prevention, San Juan, Puerto Rico

## Abstract

Puerto Rico was severely impacted by Hurricanes Irma and Maria in September 2017. The island has been endemic for dengue viruses (DENV) and recently suffered epidemics of chikungunya (CHIKV 2014) and Zika (ZIKV 2016) viruses. Although severe storms tend to increase the number of vector and nuisance mosquitoes, we do not know how they influence *Aedes aegypti* populations and arboviral transmission. We compared the abundance of female *Ae. aegypti* in autocidal gravid ovitraps (AGO traps), container habitats, and presence of RNA of DENV, CHIKV, and ZIKV in this vector before and after the hurricanes in Caguas city and in four communities in southern Puerto Rico. Two of these communities were under vector control using mass AGO trapping and the other two nearby communities were not. We also investigated mosquito species composition and relative abundance (females/trap) using Biogents traps (BG-2 traps) in 59 sites in metropolitan San Juan city after the hurricanes. Mosquitoes sharply increased 5 weeks after Hurricane Maria. Ensuing abundance of *Ae. aegypti* was higher in Caguas and in one of the southern communities without vector control. *Aedes aegypti* did not significantly change in the two areas with vector control. The most abundant mosquitoes among the 26 species identified in San Juan were *Culex* (*Melanoconion*) spp., *Culex quinquefasciatus*, *Culex nigripalpus*, and *Ae. aegypti*. No arboviruses were detected in *Ae. aegypti* following the hurricanes, in contrast with observations from the previous year, so that the potential for *Aedes*-borne arboviral outbreaks following the storms in 2017 was low.

## INTRODUCTION

Severe storms disturb aquatic habitats where mosquitoes undergo immature development and affect the spatial dispersal of both mosquitoes and vertebrate hosts, including displacement of people. New aquatic habitats for vector or nuisance mosquitoes appear after severe storms, such as the thousands of abandoned swimming pools in New Orleans following Hurricane Katrina.^1^ Increases in the relative abundance of several mosquito species that develop in groundwater habitats after hurricanes have been reported, including important vectors of arboviruses, such as West Nile, Saint Louis encephalitis, and Eastern equine encephalitis viruses.^2–8^

Communicable diseases are generally not common after natural disasters, with the exception of events resulting in the displacement of people without adequate sanitation, access to shelter, and primary health care.^9^ Nasci and Moore^4^ reviewed the impact of storms and floods in the continental United States from 1975 to 1997 and concluded that with one exception, these natural events did not increase transmission of arboviruses to humans or domestic animals. No significant increases of arboviral diseases after storms and floods were observed in other studies despite heightened mosquito abundance^2,10^ or the presence of arboviruses in mosquitoes.^11,12^ The Pan American Health Organization^13^ reported that neither dengue nor malaria cases increased over previous values after Hurricane Mitch swept through Central America. Hurricane Georges hit Puerto Rico as a category 3 hurricane in 1998 in the midst of a large dengue epidemic, yet the storm did not exacerbate the epidemic.^14^ Similarly, Hurricane Hortense hit Puerto Rico on September 10, 1996, causing heavy rains and flooding, but did not change the usual seasonal pattern of dengue transmission.^15^

Nevertheless, several reports do account for increased risk of arbovirus transmission after severe storms or flooding. Surges in West Nile neuro-invasive disease cases were reported during the 3 weeks following Hurricane Katrina in areas directly impacted by the hurricane in Louisiana and Mississippi.^16^ These authors also suggested that increased incidence of the disease the following year was possibly caused by increased exposure to mosquito bites where people had substandard living conditions and during reconstruction activities. Increased dengue and leptospirosis incidence was reported after a severe flood in southern Thailand,^17^ although the mechanism how the flood caused an increase in dengue incidence was not investigated. An association between increased dengue incidence and high river levels in Bangladesh was explained by accumulation of garbage, including plastic containers along water bodies.^18^

Although hurricanes are frequent in many tropical countries where dengue viruses (DENV) are endemic, no previous studies assessed how hurricanes affect the main dengue vector *Aedes aegypti* (L.). Here, we investigated changes in adult *Ae. aegypti* and its aquatic habitats, abundance of other urban mosquitoes, and the presence of chikungunya, dengue, and Zika viruses in *Ae. aegypti* following the impacts of category 4 Hurricanes Irma and Maria in September 2017 in Puerto Rico. Maria, which has been the third costliest hurricane in the U.S. history, traversed the island on September 20, 2017. Winds near 250 km/hours, heavy rains, and floods devastated the islands.^19^

## METHODS

We compared adult *Ae. aegypti* populations before and immediately following the hurricanes in two areas where we have been conducting vector surveillance and control activities: the city of Caguas, Caguas municipality, and four communities in southern Puerto Rico. We also initiated adult mosquito surveillance of other mosquito species, including *Ae. aegypti*, in the metro area of San Juan city 1 month after Hurricane Maria. Immature surveys in Caguas city before and after the hurricanes were conducted to understand how the storms affected the composition and abundance of containers producing *Ae. aegypti*.

### Caguas city study.

We compared female *Ae. aegypti* per trap per week in 360 surveillance autocidal gravid ovitraps (SAGO traps) placed at fixed stations throughout the city from the second week of October to December 2016 (non-hurricane year) and from the second week of October to December 2017 (hurricane year). Hurricanes Irma and Maria interrupted weekly sampling from the first week in September to the first week in October 2017. Integrated vector management, including community awareness, source reduction, larviciding, and mass trapping using three autocidal gravid ovitraps (AGO traps)per building in most of the buildings, slowly started in late November 2016 and continued through August 2017, when control traps began to be withdrawn (CDC, unpublished data). Results from that investigation are being published elsewhere (CDC, unpublished data). We conducted pupal surveys in 395 and 677 buildings in June/July 2017 (before) and November/December 2017 (after hurricanes), respectively. The sites selected for the pupal surveys were areas around SAGO traps with above average numbers of female *Ae. aegypti*. We searched for all containers with water per building to record the abundance of *Ae. aegypti* pupae and identified the most productive types of containers. Collected pupae were preserved in 80% ethanol and taken to the laboratory for identification under a stereoscopic microscope. We also calculated the house index (HI; percentage of houses with at least one container with immature *Ae. aegypti*), container index (CI; percentage of containers with water that had immatures), Breteau index (BI; no. of positive containers per 100 houses), and container Breteau index (no. of positive containers of each type per 100 houses).

### Southern Puerto Rico study.

We have been conducting *Ae. aegypti* surveillance in four communities (241–397 houses each) in Salinas and Guayama municipalities for several years.^20^ Two communities (La Margarita and Villodas) were initially treated with source reduction and larvicing, and La Margarita received three AGO traps per home in most homes in December 2011. In February 2013, we treated Villodas with three AGO traps per home and simultaneously we started monitoring two untreated, nearby communities (Arboleda and Playa) to compare *Ae. aegypti* populations with the two treated areas. Mosquito surveillance has been conducted using 28–44 SAGO traps per community since then. We compared the abundance of *Ae. aegypti* in the study areas from August 2016 through January 2017 (non-hurricane year) with data from August 2017 through January 2018 (hurricane year). Weekly sampling was interrupted for 2 weeks (last week in September and first week in October 2017) in all four areas following Hurricane Maria. Hurricane Irma did not significantly affect surveillance or control in these southern areas.

### San Juan city study.

We deployed 59 BG-2 Sentinel (BG-S) traps throughout San Juan city from October 17 to November 17, 2017. Most BG-S traps were operated for four consecutive days for one or two consecutive weeks per site. The sampling sites were selected based on a previous study in 2005, where we trapped in various types of land covers within the city (forests, high-density housing, low-density housing, non-forested vegetation, and wetlands).^21^ We did not use CDC miniature light traps as in the previous study because our CDC light traps used D-batteries, which were in short supply immediately after the hurricane, and because no dry ice was available. BG-S traps were operated with BS-lure using rechargeable batteries that we had in stock (sealed lead-acid 12V 18 ampere hour). Collected mosquitoes were transported to the laboratory and stored at −20°C until they were enumerated and identified.

### Weather.

Meteorological stations (HOBO data loggers; Onset Computer Corporation, Boume, MA) during these studies recorded rainfall, air temperature, and relative humidity in Caguas city (eight stations) and in the southern Puerto Rico communities (three stations). We calculated accumulated rainfall during the third and second weeks before mosquito sampling because rainfall during the preceding week should not contribute to new adult *Ae. aegypti* at the time of sampling. We used average temperature and relative humidity during 3 weeks before sampling because these parameters could influence the number of adult mosquitoes present at the moment of sampling, assuming that mosquitoes do not live longer than 3 weeks. To compare weather between years, we used average weekly rainfall, average daily air temperature, and average daily relative humidity. Hurricane Maria knocked down most of our weather stations, and it took us several weeks to obtain spare parts to fix them (January 2018). We obtained missing weather data from nearby weather stations that kept recording during that time in Caguas (HSWHQ ICAGUASC5 Station 18°223′N, −66°044′W), Salinas (Camp Santiago Station 18°004′N, −66.283′W), and Guayama (Jobos Bay Station 17°956′N, −66°223′W).

### Arbovirus detections in *Ae. aegypti*.

To understand if increased mosquito abundance following the 2017 hurricanes were associated with increased arbovirus transmission, we used a trioplex real time, reverse transcription polymerase chain reaction (RT-PCR) to simultaneously detect RNA of DENV, CHIKV, and ZIKV in pools (1–20 specimens per vial) of female *Ae. aegypti* collected in SAGO traps in Caguas city and in the four communities in southern Puerto Rico.^22,23^ Pools of female *Ae. aegypti* from Caguas were collected, analyzed, and compared for the following periods: October–December 2016 and October–December 2017. Pools from the four southern communities were collected, analyzed, and compared for October 2016–January 2017 and October 2017–January 2018. Data collected during 2016 on arbovirus detections in the four southern communities were recently published.^22^

### Statistical methods.

We compared females of *Ae. aegypti* per trap per week during similar periods (October–December or January) between the year before (2016) and after the hurricanes (2017) using generalized estimating equations (GEE). We used the following weather parameters as covariates: accumulated rainfall 2–3 weeks before mosquito sampling, and average temperature and relative humidity for 3 weeks, including the sampling week. A negative binomial model with log link was used for the analyses and a first-order autoregressive function as the covariance structure for repeated measures. We reported results as means and standard errors. Statistical analyses were carried out with IBM SPSS Statistics 25 software (IBM Corporation, Armonk, NY).

## RESULTS

### Caguas city.

**Figure 1.**
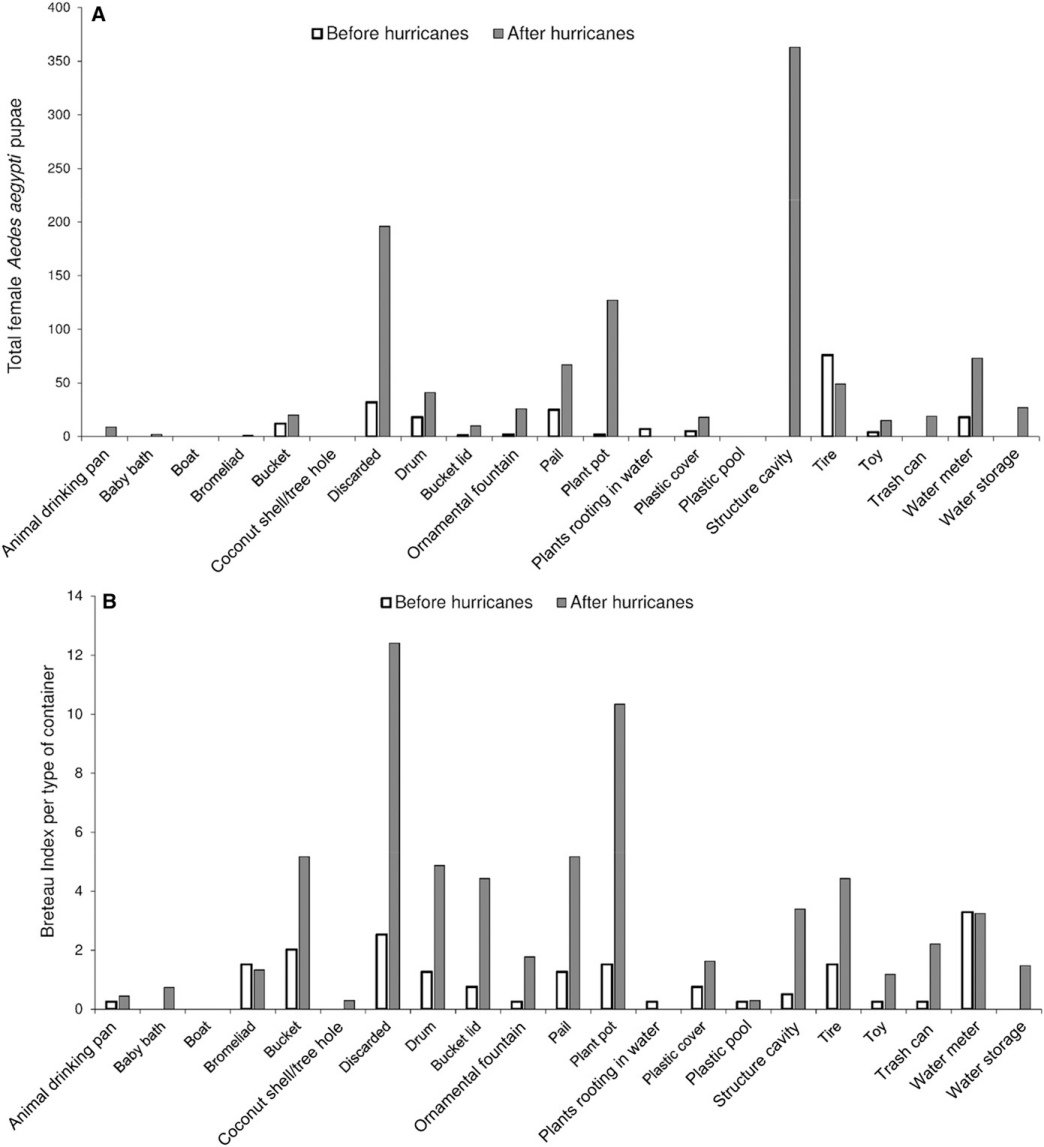
Number of female pupae of *Aedes aegypti* collected from containers in houses (**A**) and number of positive containers of each type per 100 houses (Breteau container index) (**B**) before and after Hurricanes Irma and Maria in Caguas city, Puerto Rico in 2017.

We found 441 *Ae. aegypti* pupae in 35 of 395 buildings (1.1 pupae/building) in the pupal survey before the hurricanes (June/July 2017) and 1962 pupae in 140 of 677 buildings (2.9 pupae/building) after the hurricanes (November/December 2017). The containers producing more *Ae. aegypti* pupae during the first survey were discarded tires, other discarded containers, 19-L pails, 10-L buckets, and flooded water meters (Figure 1). After the hurricanes, the most productive containers were cavities in building structures (e.g., inundated floors of houses with damaged or missing roofs, hollow bricks, and tubes), discarded containers (damaged appliances, rubble, and trash), plant pots (trivets), flooded water meters, 19-L pails, 10-L buckets, and discarded tires (Figure 1). The BI per type of container (positive containers per 100 houses) indicated that the most common containers with *Ae. aegypti* immatures before the hurricanes were flooded water meters, discarded containers, 19-L pails and 10-L buckets, discarded tires, and plant pots (Figure 1). After the hurricanes, the most common positive containers were discarded containers, plant pots, containers for water storage (drums, 19-L pails, 10-L buckets, and pail lids), water meters, and cavities in structures (Figure 1). The HI, CI, and BI (HI = 14%, CI = 3%, BI = 19) before the hurricanes were lower than those after the hurricanes (HI = 33%, CI = 12, BI = 62).

**Figure 2.**
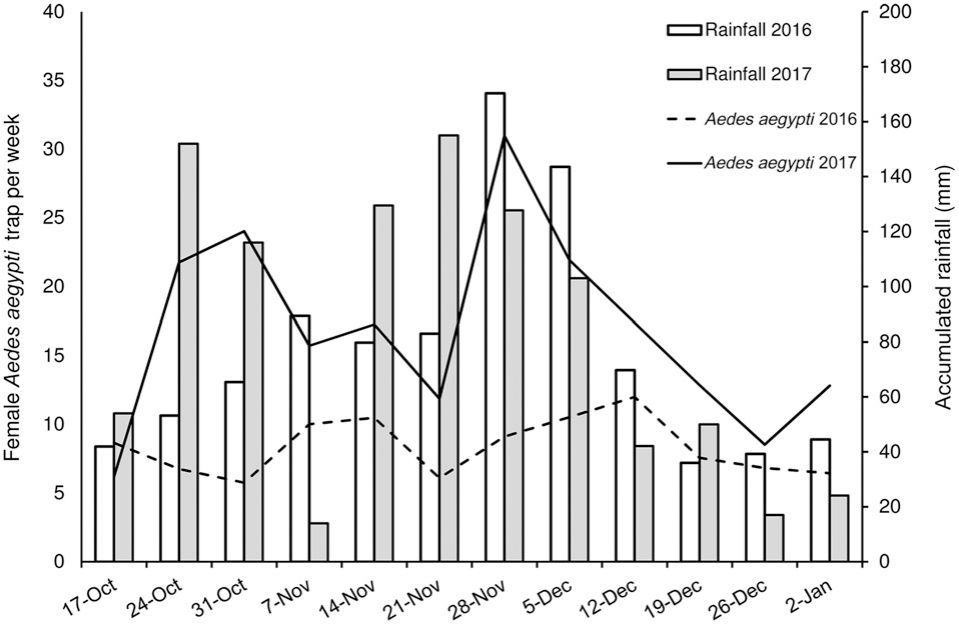
Average number of female *Aedes aegypti* per sentinel autocidal gravid ovitraps per week and accumulated rainfall (mm) during the third and second weeks before each mosquito-sampling week in 2016, and comparable results observed in 2017 after Hurricanes Irma and Maria in Caguas city, Puerto Rico. Rainfall was lag-forwarded 1.5 weeks to facilitate visual comparisons with mosquito numbers.

The average number of female adults of *Ae. aegypti* per AGO trap per week after the hurricanes in October–December 2017 (16.8 ± 0.2; *N* = 4,242 trap × weeks) was significantly different (Wald χ_1_^2^ = 220; *P* < 0.001) from the previous, non-hurricane year (October–December 2016; 8.4 ± 0.1; *N* = 4,309). There were significant effects of the covariates: accumulated rainfall (Wald χ_1_^2^ = 380; *P* < 0.001), average temperature (Wald χ_1_^2^ = 30; *P* < 0.001), and average relative humidity (Wald χ_1_^2^ = 24; *P* < 0.001). The number of female mosquitoes peaked in October and by the end of November 2017 (Figure 2). The relative abundance of *Ae. aegypti* (1.4–2.1 ± 0.04; CDC, unpublished data) prevailing in Caguas city a few months (May–August 2017) before removing the control traps in August–September 2017 was several times lower than that observed in October–December 2017 (16.8 ± 0.2), showing that the population of *Ae. aegypti* rapidly recovered after removing control measures and following the hurricanes.

Average weekly rainfall from the second week in October–December 2016 (37.8 ± 8.1 mm) was similar to that registered for the same time interval in 2017 (41.5 ± 9.1; Figure 2) in Caguas city. Weekly rainfall distribution varied between years, with one peak in November 2016 and two peaks in October and November 2017 (Figure 2). Average temperature and relative humidity in 2016 (25.9 ± 0.3°C; 85.2 ± 0.6%) and 2017 (26.2 ± 0.2°C; 80.8 ± 1.1%) were comparable.

We found one DENV-, three CHIKV-, and 44 ZIKV-positive pools of female *Ae. aegypti* in October–December 2016, the year of the Zika epidemic in Puerto Rico (4,385 pools). No arboviruses were detected in *Ae. aegypti* in Caguas city after the hurricanes in October–December 2017 (5,257 pools).

### Southern Puerto Rico.

**Figure 3.**
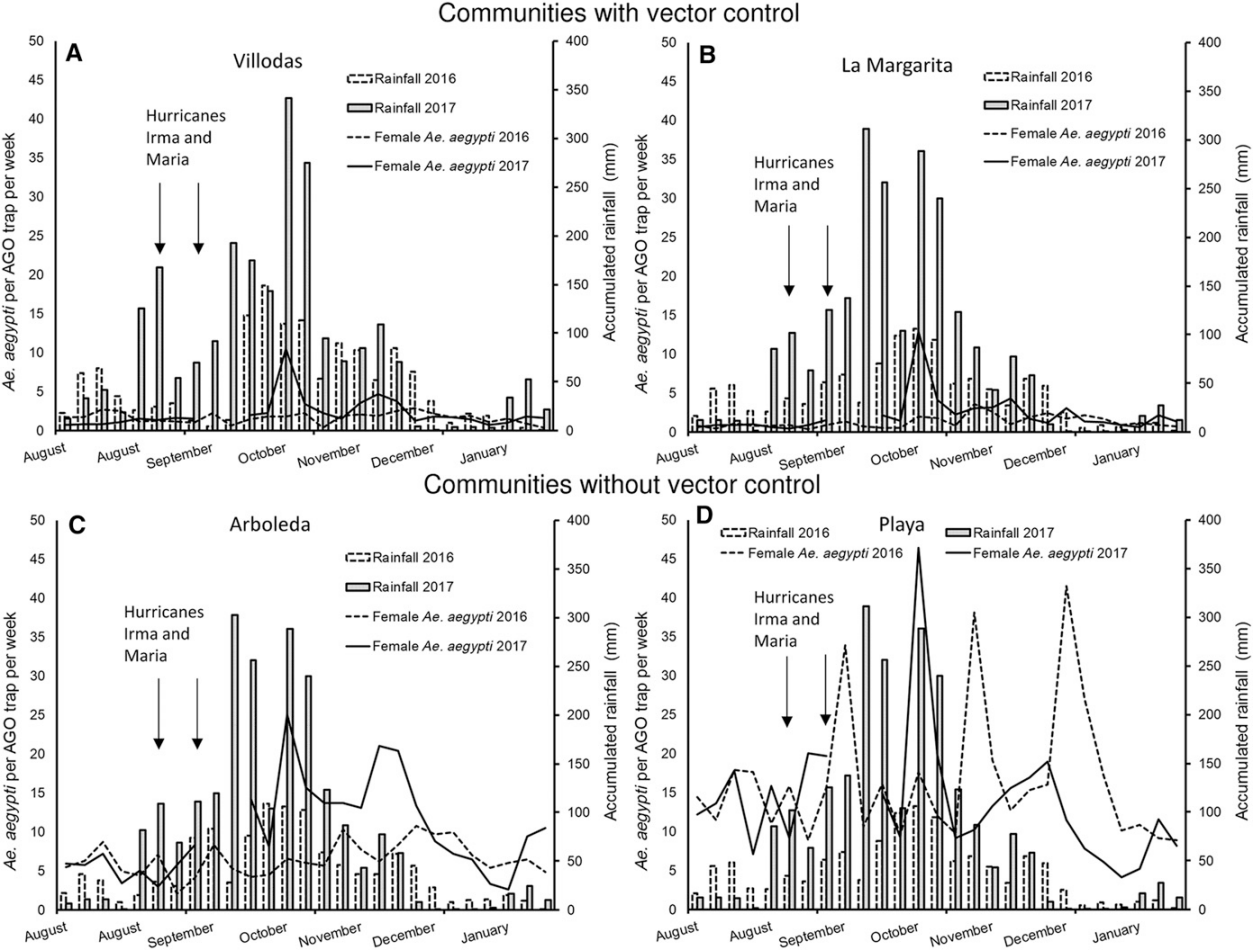
Comparing average numbers of female *Aedes aegypti* and accumulated rainfall (mm) during the third and second weeks before each mosquito-sampling week in four communities in southern Puerto Rico. Two communities (**A** and **B**) were under vector control interventions using three autocidal gravid ovitraps per house in most houses in 2016 and in 2017 when the Hurricanes Irma and Maria hit Puerto Rico. The other two communities (**C** and **D**) were nearby sites without vector-control interventions. Rainfall was forwarded 1.5 weeks to facilitate comparisons with mosquito numbers.

Hurricane Maria directly affected all four study sites, bringing down power lines and trees, and partially or totally destroying many buildings. Yet, few control AGO traps were lost (20–37) in the two communities under mass trapping, but many were toppled or dry (95 of 498 in Villodas and 145 of 568 in La Margarita). There were simultaneous sharp increases in *Ae. aegypti* in all four communities 5 weeks after Hurricane Maria (Figure 3). Average *Ae. aegypti* abundance at each of the four sites during the fifth week after the hurricane was the largest ever recorded for those sites since 2013 (CDC, unpublished data). Abundance of *Ae. aegypti* in the two communities with control AGO traps (Villodas and La Margarita) has been small and steady for several years,^22^ at or below two females per trap per week. These low numbers were observed for most of 2016/2017 and 2017/2018 (Figure 3A and B), except following Hurricane Maria when abundance increased to 10–14 mosquitoes per trap per week. Despite these temporary abundance peaks, the GEE analyses showed lack of significant differences (Wald χ_1_^2^ = 0.4; *P* > 0.05) in the average abundance of *Ae. aegypti* per trap per week for the months of the study between years (non-hurricane 1.7 ± 0.1 versus hurricane 2.21 ± 0.1) and a significant effect of rainfall (Wald χ_1_^2^ = 88.4; *P* < 0.001) in Villodas. Similarly, average mosquito numbers in La Margarita did not significantly (Wald χ_1_^2^ = 0.4; *P* < 0.001) change between years (1.3 ± 0.1 versus 2.2 ± 0.1). Covariates rainfall (Wald χ_1_^2^ = 155.9; *P* < 0.001) and temperature (Wald χ_1_^2^ = 117.6; *P* < 0.001) were significant.

Mosquito abundance after the hurricanes in one of the two communities without vector control (Arboleda 10.0 ± 0.3 females/trap/week; Figure 3C) was steadily more than observed levels in the previous year from October to December (6.5 ± 0.2). The GEE analysis showed significant differences between years (Wald χ_1_^2^ = 39.1; *P* < 0.001) and significant effects of rainfall (Wald χ_1_^2^ = 155.3; *P* < 0.001) and temperature (Wald χ_1_^2^ = 6.6; *P* < 0.05). Average mosquito abundance in the other community without vector control (Playa 13.8 ± 0.6) was actually lower than in the previous, non-hurricane year (16.6 ± 0.6). Results from the GEE analysis showed that those differences were significant (Wald χ_1_^2^ = 20.0; *P* < 0.001) despite the fact that the peak in mosquito abundance that was observed 5 weeks after the hurricane reached its largest value ever (42 females/trap/week; Figure 3D). Effects of covariates rainfall (Wald χ_1_^2^ = 122.6; *P* < 0.001) and temperature (Wald χ_1_^2^ = 29.1; *P* < 0.001) were significant.

Average weekly rainfall for all sites after the hurricanes from August 2017 to January 2018 (40.4 ± 6.5 mm) was double than that registered for August 2016–January 2017 (19.5 ± 2.5; Figure 3). Overall, average temperature and relative humidity in August 2016–January 2017 (26.9 ± 0.2°C; 76.3 ± 0.5%) and August 2017–January 2018 (26.8 ± 0.1°C; 76.5 ± 0.6%) were similar.

No pools of *Ae. aegypti* females tested positive for DENV, CHIKV, or ZIKV in either August 2016–January 2017 or August 2017–January 2018 in the two communities treated with AGO traps (La Margarita 213 pools and Villodas 148 pools). We found five and eight positive pools of female *Ae. aegypti* with ZIKV in the untreated communities (Arboleda 265 pools and Playa 626 pools, respectively), in August 2016–January 2017, and no arboviruses were detected in *Ae. aegypti* from these communities in August 2017–January 2018 (382 and 480 pools, respectively). Thus, no arbovirus activity was evident in *Ae. aegypti* in any of these communities following Hurricanes Irma and Maria in 2017.

### San Juan city.

**Figure 4.**
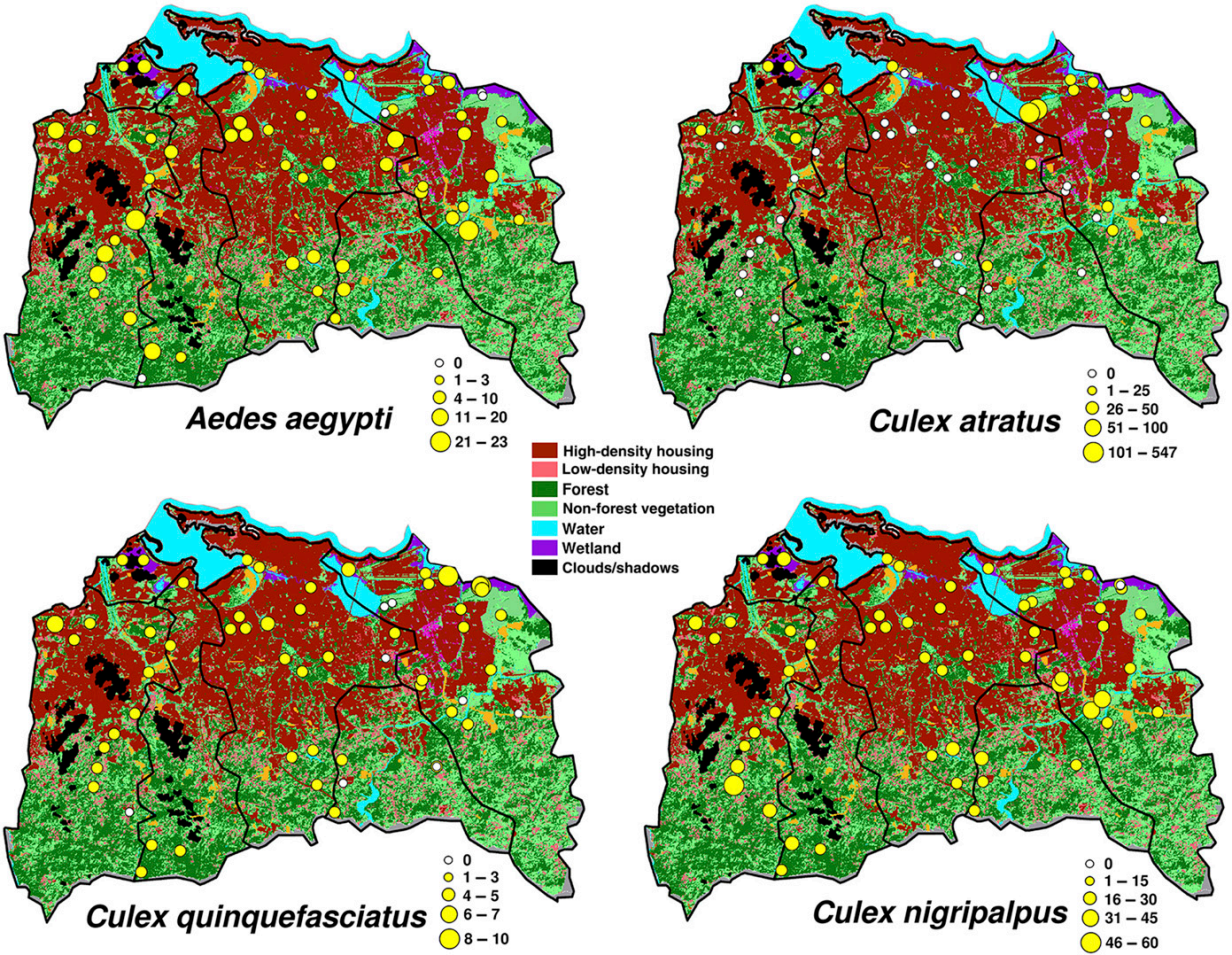
Map of the metropolitan area of San Juan city showing the average number of mosquitoes per BG-S trap per day of the four more abundant mosquito species across major landscape elements (forests, high-density housing, low-density housing, non-forested vegetation, and wetlands) after Hurricanes Irma and Maria hit Puerto Rico in 2017.

We placed BG-S traps at 59 locations in the following municipalities of the metro area of San Juan city: Bayamón, Carolina, Cataño, Guaynabo, San Juan, and Trujillo Alto (Figure 4). We collected 11,797 females of 26 species of mosquitoes in 451 trap days (Table 1). There were 17 trap failures, including some stolen batteries or traps, wire problems, and ants. The most common female adult mosquito species in the traps were *Culex atratus*, *Culex quinquefasciatus*, *Ae. aegypti*, and *Culex nigripalpus*. More *Cx. atratus* were captured in non-forest vegetation areas and in forests. *Culex quinquefasciatus* was more abundant in wetland areas followed by low-density housing areas. *Aedes aegypti* was more common in low-density housing areas, but like *Cx. quinquefasciatus*, this species was widely distributed across landscapes in the metro area. *Culex nigripalpus* was more abundant in wetlands and forests (Table 1). The average numbers of *Ae. aegypti* per trap per day in the various landscapes were as follows: low-density housing 9.8 ± 1.1, high-density housing 3.4 ± 0.4097, non-forest vegetation 2.7 ± 0.5, forests 2.6 ± 1.0, and wetlands 2.1 ± 0.3.

**Table 1:** Composition and abundance of female adult mosquitoes collected in BG-S traps in the metro area of San Juan city, Puerto Rico, after Hurricanes Irma and Maria (October–November 2017)

Mosquito species	Forest	High-density housing	Low-density housing	Non-forest vegetation	Wetlands	Total
*Culex (Mel.) atratus* Theo.	1.302	1	11	3.884	137	5.335
*Culex (Cux.) quinquefasciatus* Say	559	598	873	594	1.081	3.705
*Aedes (Stg.) aegypti* (L.)	203	300	839	213	211	1.766
*Culex (Cux.) nigripalpus* Theo.	92	56	73	85	304	610
*Uranotenia (Ura.) lowii* Theo.	12	1	0	7	62	82
*Culex (Mel.) erraticus* (Dyar and Knab)	0	1	3	15	28	47
*Culex (Mca.) antillummagnorum* Dyar	2	0	9	30	0	41
*Aedes (Gym.) mediovitattus* (Coquillett)	10	1	6	5	10	32
*Culex (Cux.) secutor* Theo.	7	0	0	2	19	28
*Anopheles (Ano.) vestitipennis* Dyar and Knab	0	0	0	0	22	22
*Culex* spp.	3	7	3	4	5	22
*Anopheles (Ano.) grabhamii* Theo.	0	0	0	0	16	16
*Unotenia (Ura.) sociales* Theo.	2	0	0	2	12	16
*Culex (Mel.) iolambdis* Dyar	1	0	1	5	6	13
*Mansonia (Man.) dyari*	0	0	0	2	10	12
*Culex (Cux.) janitor* Theo.	1	1	1	4	4	11
*Culex (Cux.) chidesteri* Dyar	0	0	0	0	9	9
*Unotenia (Ura.) cooki* Root	1	0	1	1	5	8
*Culex (Dei.) magnus* (Theo.)	0	0	0	0	7	7
*Aedes (Och.) tortilis* (Theo.)	0	0	0	4	0	4
*Anopheles (Ano.) atropos* Dyar and Knab	0	0	0	0	3	3
*Culex (Mel.) peccator* Dyar and Knab	1	2	0	0	0	3
*Psorophora (Gra.) jamaicensis* (Theo.)	0	0	0	1	1	2
*Anopheles (Nys.) albimanus* Wiedemann	0	0	0	0	1	1
*Culex (Cux.) habilitator* Dyar and Knab	0	0	0	0	1	1
*Culex (Mel.) taeniopus* Dyar and Knab	0	0	1	0	0	1
Total	2.196	968	1.821	4.858	1.954	11.797

## DISCUSSION

Severe storms are expected to increase the number of mosquitoes by increasing or decreasing the edge surface of groundwater habitats, filling cavities/containers with rainwater, and generating additional container aquatic habitats in destroyed or abandoned properties (damaged appliances, inundated floors/roofs, and rubble). The latter two events are relevant to the ecology of *Ae. aegypti* because this species does not use groundwaters but used water-filled containers for immature development. This study showed large numbers of containers and pupae of *Ae. aegypti* in the weeks following Hurricane Maria in Caguas city, including highly productive, flooded floors and rubble resulting from wind/flood damage. Adult female of *Ae. aegypti* doubled in numbers after the hurricanes in this city. Surprisingly, the amount of rainfall from October to December in 2016 and 2017 was similar in Caguas, which may indicate that the increase in the number of adult mosquitoes was mainly a result of more containers holding water. Year 2016 was a rather wet year itself.^22^

The numeric response of the *Ae. aegypti* population to increased rainfall in 2017 as compared with 2016 in southern Puerto Rico was heterogeneous, although mosquito numbers in all four study sites sharply increased exactly 5 weeks after Hurricane Maria. The only significant average increase in adults of *Ae. aegypti* from October 2017 through January 2018 in comparison with the same period in 2016/2017 was observed in one of the two locations without vector control. The other location without vector control actually showed significantly lower mosquito numbers than in the previous year when rainfall was about half. Contributing to the heterogeneity of responses, the two sites with vector control (three AGO traps per home in most homes plus surveillance AGO traps) did not show any significant difference in mosquito captures in comparison with the previous year. We were able to continue vector surveillance in the four southern areas 3 weeks after Maria and to reset or replace lost AGO control traps within 5 weeks in the two communities with vector control, despite pervasive limited living conditions at the time. Wherever significant increases in *Ae. aegypti* captures were observed after the hurricanes, they returned to pre-hurricane levels during December 2017 or shortly thereafter.

Although monitoring unusual increases in mosquitoes after natural disasters is important, some areas may already contain enough mosquitoes to cause local arbovirus outbreaks without any additional increases caused by the storms. For example, average *Ae. aegypti* captures before or after the hurricanes in Caguas city and in the two sites without vector control in southern Puerto Rico were several times higher than two female *Ae. aegypti* per trap per day; the relative abundance associated with 50% less prevalence of anti-chikungunya IgG antibodies in areas with mass-trapping vector control.^24^

Whether vector control needs to be applied after natural disasters, such as after severe storms, should be decided based on assessments of abundance of key vector or nuisance mosquito species, virus infection rates in mosquitoes, and seroprevalence in sentinel or wild vertebrate hosts.^4^ Emergency vector control has been practiced in the United States after severe storms due to significant increases in ground-water mosquitoes.^3,5,6,8^ Our study of mosquito captures in the metro area of San Juan could not be directly compared with a previous study^21^ because we did not have the same traps available after the hurricanes. However, we obtained similar results to the previous study conducted during a wetter than normal year (2005) when we used CDC miniature light traps with CO_2_: high numbers of *Culex* (*Melanoconion*) spp. mosquitoes in less disturbed areas with tall or short vegetation and wetlands, 26 mosquito species versus 28 in 2005, and widespread distribution of *Cx. nigripalpus*; an important West Nile virus vector.^25^ The two other widespread species found were *Ae. aegypti* and *Cx. quinquefasciatus*, both of which were more abundant in areas with low-density housing. People in the metro area were mostly exposed to these urban mosquito species and to a lesser extent to *Cx. nigripalpus*. Because of lack of power, piped water, damaged houses, and reconstruction activities, the people in Puerto Rico were exposed to increased mosquito bites after the hurricanes.

Dengue, chikungunya, and Zika viruses were not detected in *Ae. aegypti* in Caguas city or in the four southern communities after the hurricanes. In contrast, we detected all of these viruses in *Ae. aegypti* the previous year in Caguas city and in two of the communities without vector control in southern Puerto Rico. No viruses were found during comparable periods before or after the hurricanes in the two communities under vector control with mass AGO trapping. Because *Ae. aegypti* adults do not fly far, the presence of arboviruses in these mosquitoes indicates the presence of local infectious persons.^26^ Our results suggest that these arboviruses were not being actively transmitted in the study areas. Lack of arboviruses in *Ae. aegypti* after the hurricanes in 2017 is consistent with the paucity of human cases during the same periods. For example, less than 20 Zika cases per week were reported from Puerto Rico from October 2017 to January 2018, whereas up to 1,000 cases per week were reported during the same time period during the Zika outbreak in 2016/2017.^27,28^ Thus, the potential for outbreaks after the hurricanes was low despite the abundant populations of *Ae. aegypti* even in the areas where we detected significant increases in their abundance.
